# Understanding *Alstroemeria pallida* Flower Colour: Links between Phenotype, Anthocyanins and Gene Expression

**DOI:** 10.3390/plants10010055

**Published:** 2020-12-29

**Authors:** Amanda Donoso, Constanza Rivas, Alan Zamorano, Álvaro Peña, Michael Handford, Danilo Aros

**Affiliations:** 1 Faculty of Agricultural Sciences, University of Chile, Santiago 8820808, Chile; amandadonoso@uchile.cl (A.D.); constanzarivas@u.uchile.cl (C.R.); agezac@u.uchile.cl (A.Z.); apena@uchile.cl (Á.P.); 2 Department of Biology, Faculty of Sciences, University of Chile, Santiago 7800003, Chile; mhandfor@uchile.cl

**Keywords:** plant breeding, ornamental plant, real-time PCR, HPLC, colour chart

## Abstract

Flower colour is mainly due to the accumulation of flavonoids, carotenoids and betalains in the petals. Of these pigments, flavonoids are responsible for a wide variety of colours ranging from pale yellow (flavones, flavonols and flavanodiols) to blue-violet (anthocyanins). This character plays a crucial ecological role by attracting and guiding pollinators. Moreover, in the ornamental plants market, colour has been consistently identified as the main feature chosen by consumers when buying flowers. Considering the importance of this character, the aim of this study was to evaluate flower colour in the native Chilean geophyte *Alstroemeria pallida*, by using three different approaches. Firstly, the phenotype was assessed using both a colour chart and a colourimeter, obtaining CIELab parameters. Secondly, the anthocyanin content of the pigmented tepals was evaluated by high-performance liquid chromatography (HPLC), and finally, the expression of two key flavonoid genes, *chalcone synthase* (*CHS*) and *anthocyanidin synthase* (*ANS*) was analysed using real-time polymerase chain reaction (PCR). Visual evaluation of *A. pallida* flower colour identified 5 accessions, ranging from white (Royal Horticultural Society (RHS) N999D) to pink (RHS 68C). Moreover, this visual evaluation of the accessions correlated highly with the CIELab parameters obtained by colourimetry. An anthocyanidin corresponding to a putative 6-hydroxycyanidin was identified, which was least abundant in the white accession (RHS N999D). Although *CHS* was not expressed differentially between the accessions, the expression of *ANS* was significantly higher in the accession with pink flowers (RHS 68C). These results suggest a correlation between phenotype, anthocyanin content and *ANS* expression for determining flower colour of *A. pallida*, which could be of interest for further studies, especially those related to the breeding of this species with ornamental value.

## 1. Introduction

The accumulation of flavonoids, carotenoids and betalains, is the principal determinant of flower colour [1]. Flavonoids represent the most important group with more than 8000 structures reported [2] and their accumulation in petals is responsible for a wide variety of colours, ranging from orange, red, magenta, violet and blue (anthocyanins) to colourless or very pale yellow (flavones and flavonols) [3]. Such pigments have been characterised in species such as *Dahlia variabilis* [4], *Rosa × hybrida* [5], *Matthiola incana* [6] and *Paeonia lactiflora* [7]. Flavonoid biosynthesis has been well studied, and several genes codifying for enzymes involved in the metabolic pathway of anthocyanins have been described and characterised, such as chalcone synthase (*CHS*) and anthocyanidin synthase (*ANS*) [3,8]. Furthermore, terminal modification by the addition of sugars, methyl, ferulate and other groups produce an even greater variety of different anthocyanins, and thus colours [9]. Light, temperature, pH, oxidising and reducing agents can significantly affect the stability of anthocyanins [10], while co-pigments, vacuolar pH and chelation are important factors that also alter flower colour produced by anthocyanins [11].

The importance of flower colour is clearly ecological as this trait has been widely described as the main cue to guide and attract pollinators [12]. Even though several stimuli have been described as part of the pollination syndrome, the foraging behaviour of pollinators relies particularly on colour [13] over other stimuli such as floral scent [14].

In addition to the crucial role flower colour plays during pollination, this character is also very important for the market of ornamental plants where it is one of the most appreciated attributes chosen by consumers when buying flowers [15,16]. Normally, specific flavonoids are associated with each flower, which means that a limited range of flower colours is available within a species due to genetic restrictions. Thus, many breeding programmes have focused on flower colour as the main selection criteria for generating and releasing new cultivars, especially by means of hybridisation and mutation [17], but also by genetic engineering [11].

One of the most important ornamental species in the market of ornamental plants, both as a cut flower and pot plant, is alstroemeria, which has been bred from the South American native species to obtain new cultivars [18]. Native alstroemeria species display a wide variety of flower colour including yellow (*Alstroemeria aurea*), pink (*A. pelegrina*), orange (*A. ligtu* spp. *ligtu*), white (*A. pulchra*) and violet (*A. violacea*) [19]. The anthocyanin composition of alstroemeria flowers has been characterised using high-performance liquid chromatography (HPLC), resulting in the reporting of 3-rutinosides of 6-hydroxydelphinidin, 6-hydroxycyanidin, cyanidin, delphinidin, and 3-glycosides of cyanidin and delphinidin as the major anthocyanins present [20,21,22]. However, no studies related to the expression of genes associated with the biosynthesis of these anthocyanins have been reported in alstroemeria, or in its cultivars.

Considering the importance of flower colour for the market of ornamental plants and intraspecific biodiversity in terms of colour preliminarily observed in *A. pallida* [23], a Chilean native species from the Andes mountains, we characterised the flower colour of 5 accessions of this species considering phenotype, anthocyanin content and the expression of key genes associated with the biosynthesis of these compounds. Furthermore, integration of the data obtained was performed in order to better understand flower colour in alstroemeria.

## 2. Results

### 2.1. Phenotypic Characterisation

Five accessions were distinguished according to tepal colour using the RHS mini colour chart (The Royal Horticultural Society and Flower Council Holland, London, UK), including white (RHS N999D and RHS 155B), light pink (RHS 56C and RHS 65B) and pink (RHS68C) varieties. These accessions showed significant differences in terms of the three CIELab parameters evaluated. Thus the pink accession (RHS 68C) possessed a C* value that is significantly higher (22.02) compared to the other accessions, which means that it has the highest flower colour intensity or saturation of the accessions analysed. In terms of h*, RHS N999D had the highest value (72.55°), which is closer to yellow (90°) while RHS 68C showed the lowest value (12.54), closer to red (0°). The lowest value of L* was observed in the pink accession (76.26), meaning that the flower colour of this accession was darker in comparison with the others. No significant differences for L* were observed between the remaining four accessions (Table 1).

The correlations calculated between the variables evaluated for colour characterisation were all significant (*p* ≤ 0.05), including positive correlations between L*/h* and C*/VCI, and negative correlations between L*/C*, L*/VCI, C*/h* and h*/VCI (Table 2).

### 2.2. Anthocyanin Characterisation

In all *A. pallida* accessions, one anthocyanin compound was detected by HPLC analysis, with a retention time of 10.00 min and an absorption spectrum with peaks (λ_max_) at 518 nm and 282 nm. These characteristics are consistent with a putative 6-hydroxycyanidin. This anthocyanidin has been previously reported in other species of alstroemeria [24] and corresponds to a cation that has a hydroxy group substituted at position 6 of cyanidin. It is derived from a cyanidin cation and its structure is found rarely in nature but has been determined in several alstroemeria cultivars [25].

**Figure 1:**
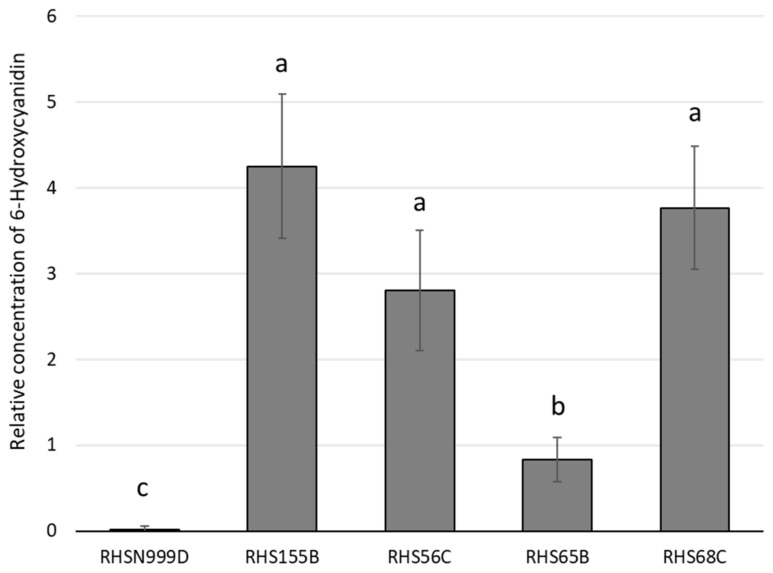
Relative concentration of a putative 6-hydroxycyanidin (eq. malvidin 3-glucoside/FW [g]) observed in the tepals of 5 *Alstroemeria pallida* accessions. Different letters indicate significant differences in ANOVA (*n* = 4) followed by an least significant difference (LSD) test (*p* ≤ 0.05, mean ± SE).

The relative concentration of this putative 6-hydroxycyanidin in the tepals of the white *A. pallida* accession RHS N999D was lower and almost insignificant (=0.01) in comparison with the other four accessions. Consistently, RHS 155B, RHS 56C and RHS 68C showed higher concentrations of 6-hydroxycyanidin with values of 4.25, 2.80 and 3.77, respectively (Figure 1). Finally, no signifcant differences were observed in the tepal cell pH measured in each accession (Appendix A), therefore the anthocyanin content observed was not affected by cellular pH.

### 2.3. Relative Expression of Chalcone Synthase (CHS) and Anthocyanidin Synthase (ANS)

**Figure 2:**
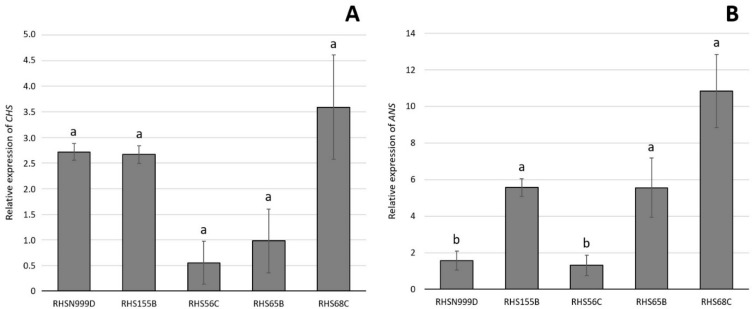
Relative expression of *chalcone synthase* (*CHS*) (**A**) and *anthocyanidin synthase* (*ANS*) (**B**) evaluated by real-time quantitative polymerase chain reaction (qPCR) in tepals of 5 *Alstroemeria pallida* accessions (± standard error (SE), *n* = 12). Results are shown as expression relative to the housekeeping gene *eEF1A*. Different letters indicate significant differences using ANOVA followed by an LSD test (*p* ≤ 0.05).

**Figure 3:**
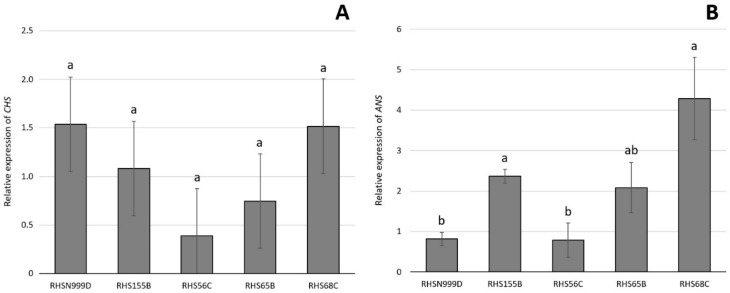
Relative expression of *CHS* (**A**) and *ANS* (**B**) evaluated by real time qPCR in tepals of 5 *Alstroemeria pallida* accessions (±SE, *n* = 12). Results are shown as expression relative to the housekeeping gene *18S rRNA*. Different letters indicate significant differences using ANOVA followed by an LSD test (*p* ≤ 0.05).

The relative expression of *CHS* was not significantly different between the five *A. pallida* accessions when using either *eEF1A* (Figure 2A) or *18S rRNA* (Figure 3A) as housekeeping genes. Relative gene expression values ranged between 0.98 and 2.72 compared to *eEF1A* and between 0.75 and 1.54 compared to *18S rRNA*.

However, the relative expression of *ANS* was significantly different between the white accession (RHS N999D) and the pink accession (RHS 68C) when using both *eEF1A* (Figure 2B) and *18S rRNA* (Figure 3B) as housekeeping genes. There were no significant differences between the pink accession and the other white accession (RHS 155B) and one of the light pink (RHS 65B) accessions (Figure 2B and Figure 3B).

Nucleotide sequence of the amplicons obtained for both genes were identical among the different accessions analysed and also with *A. pelegrina* transcriptome sequences. BLASTn comparison showed a maximum identity of 80.2% with the predicted sequence of *CHS2* gene from *Rhodamnia argentea* (Accession number XM_030693740) for *CHS* amplification product and 86.4% of nucleotide identity for *Lonicera caerulea ANS* gene (Accession number KT362346) for *ANS* amplicon.

### 2.4. Principal Component Analysis

**Figure 4:**
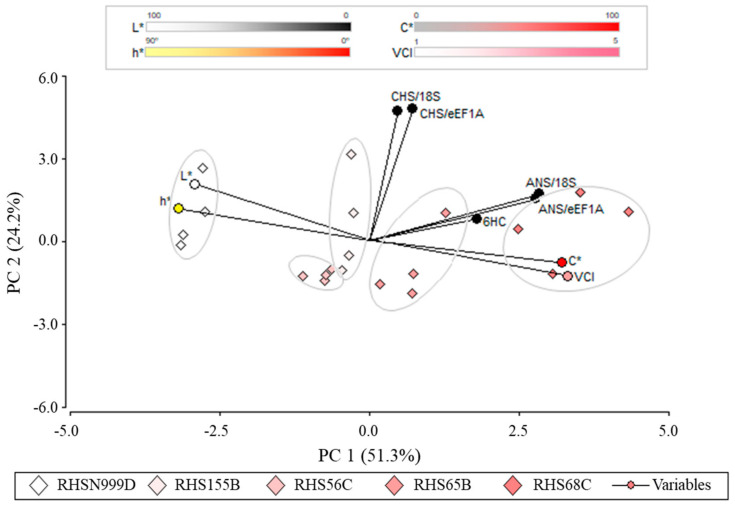
Biplot analysis of the principal components of the variables L*, C*, h*, VCI, 6-hydroxycyanidin relative concentration, *CHS* and *ANS* relative expression and the 5 *A. pallida* accessions de *A. pallida*. PC1: Principal component 1. PC2: Principal component 2.

In order to determine correlations that underlie the phenotypic, biochemical and molecular characteristics of the 5 contrasting accessions, a principal component analysis (PCA) was performed. Two main principal components (PC) accounted for 75.5% of the total variability: PC1 (51.3%) and PC2 (24.2%). PC1 is mainly responsible for the difference between the CIELab parameters, anthocyanin content and *ANS* expression, while PC2 relates C* and VCI against *ANS* expression. In general, accessions with lower VCI (i.e., RHS N999D) have flowers with a lighter colour (L*), a higher yellowness (h*), a lower colour saturation (C*), a lower relative concentration of 6-hydroxycyanidin and a lower relative expression of *ANS*. Exactly the opposite was found for accessions with a higher VCI (i.e., RHS 68C). *CHS* expression did not correlate with the other variables evaluated (Figure 4).

Correlations calculated between CIELab parameters (C*, L*, h* and VCI), 6-hydroxycyanidin content and gene expression showed 19 significant correlations (*p* ≤ 0.05) (Table 3). VCI showed high significant correlations with the CIELab parameters; negatively with L* (−0.81) and h* (−0.89), and positively with C* (0.92). Strong correlations were also observed between CIELab parameters, L* and h* (0.74), L* and C* (−0.75), and C* and h* (−0.72). 6-hydroxycyanidin content showed significant correlations with *ANS* ref. 18S (0.45) and h* (−0.54). Finally, an expected high and positive correlation was observed for *CHS* expression between normalisations against both *18S* and *eEF1A* (0.80).

## 3. Discussion

### 3.1. Phenotypic Characterisation of Flower Colour

Different accessions of *A. pallida* have a variety of colours, ranging from white to pink. High heterozygosity produced by cross pollination mediated by insects has been observed in the *Alstroemeria* genus [26], which could explain the greater variety of flower colour in this species [27], probably due to intra and even inter specific hybridisation. These five accessions were sampled from a population habiting a restricted area (approximately 5000 m^2^) so considering the prevalence of natural pollinators, the possibilities of hybridisation are quite high. Intraspecific flower variation has been previously studied and related to flavonoid patterns within species, which apparently occurs randomly in the plant kingdom [28]. More recent examples have also been reported and discussed in orchids [29] and *Mimulus lewisii* [30]. Furthermore, flower colour polymorphism may have some ecological consequences in natural populations in terms of the behaviour of some pollinators [31] and the frequency of herbivore damage [32].

The visual categorisation performed using the RHS colour chart (Figure 1) correlated well with the quantitative parameters (L*, C* and h*) (Table 1). Thus, the RHS N999D accession, which is visually the palest, has a high luminosity (L* close to 100), low saturation (C* close to 0) and a more yellowish hue (h* close to 90°). On the other hand, the RHS 68C accession, which has the most intense colour, shows low luminosity (L* close to 0), high saturation (C* close to 100) and a redder hue (h* close to 0°). The negative correlation between the L* and C* parameters observed among the different accessions has been previously reported in flowers of *Paeonia lactiflora* [33] and *Lycoris longituba* [34].

### 3.2. Anthocyanin Content in Tepals

Several anthocyanins have been reported in alstroemeria, including glycoside 6-hydroxycyanidins in red flowers [24,25], glycoside 6-hydroxydelphinidins in red-violet flowers [20], and 6-hydoxypelargonidin 3-rutinoside and 6-hydoxypelargonidin 3-glucoside in red-orange cultivars [22]. The anthocyanin content of Chilean native alstroemerias, including *A. pallida*, was analysed reporting a high relative concentration of 3-rutinoside cyanidin, intermediate relative concentrations of 6-hydroxycyanidin 3-rutinoside and traces of acylated 6-hydroxycyanidin 3-rutinoside and 3-malonylglucoside cyanidin in this species [35]. In contrast, in this study, we identified a peak with the characteristics of 6-hydroxycyanidin glycoside. This compound corresponds to an anthocyanin linked to an unknown sugar. This suggests that alstroemeria flowers harbour a variety of glycosylated pigments that provide different colours, including those described in the study mentioned above [35].

The relative concentration of this putative 6-hydroxycyanidin in each of the accessions, showed that it was virtually absent in the white accession (RHS N999D) (Figure 1), which could explain its lack of colouration. This type of floral colour polymorphism is common, where the absence of flower pigment production results in the appearance of a white morphotype [36], as described in several species, such as *Digitalis purpurea*, *Echium plantaginetum*, *Phlox drummondii* and *Phlox pilosa* [37] and more recently in *Wahlenbergia albomarginata* [38], *Hesperis matronalis* [39] and *Mimulus lewisii* [30].

A clear trend towards an increase in the relative concentration of 6-hydroxycyanidin alongside an increase in colouration was not observed (Figure 1). Furthermore, the relative concentration of putative 6-hydroxycyanidin was inversely correlated with the hue (h*) of the accessions but did not correlate with luminosity or colour saturation (Table 3). That is, accessions with a higher relative concentration of 6-hydroxycyanidin have a more reddish colour, but do not necessarily have a higher colour saturation. These results could be explained by the differential presence of as-yet undetected or unidentified flavones and flavonols in these accessions, as such compounds can act as co-pigments [40]. Such weakly-coloured substances may bind stably to anthocyanins to reinforce their colour, making it more intense. Although the concentration of these co-pigments in the accessions was not determined in this research, when observing the chromatogram produced by HPLC at 280 nm (Appendix B), a large amount of low molecular weight phenolic compounds was observed, which may contribute to the stability of anthocyanins and, therefore, to the development of colour in this species. In the future, a more sensitive method to assess flower colour such as high-performance liquid chromatography-mass spectrometry (HPLC-MS) [41] or ultra high-performance liquid chromatography (UPLC-MS) [42] will allow a more precise identification and characterisation of these compounds, which is very important to fully understand the process of flower pigmentation.

### 3.3. Relative Expression of CHS and ANS

The anthocyanin biosynthetic pathway has been widely studied in ornamental [7,43] and model species [44]. The pathway is composed of four stages, involving 12 gene products [45], of which *CHS* and *ANS*, amplified and analysed in this study, are key. *CHS* participates at the beginning of the pathway, catalysing the condensation of three molecules of malonyl-CoA with one molecule of p-coumaroyl-CoA to produce one molecule of 4′,2′,4′,6′-tetrahydroxychalcone (chalcone), a key intermediate in the formation of flavonoids. On the other hand, *ANS* is involved towards the end of the anthocyanin biosynthetic pathway, catalysing the transformation of leucoanthocyanidins into anthocyanidin [1].

The relative expression of *CHS* remained constant, showing no significant differences between the five *A. pallida* accessions assessed in this study (Figure 3 and Figure 4), suggesting that the differences in colour observed between the accessions analysed are probably not due to the expression of this gene. On the other hand, *ANS* showed a differential expression pattern among the accessions, and was significantly lower in the white (RHS N999D) accession compared to the pinker ones (RHS 65B and RHS 68C) (Figure 2). This expression pattern partially coincides with the concentration of the putative 6-hydroxycyanidin (Table 3), suggesting that *ANS* is involved in the accumulation of this pigment in *A. pallida* flowers. This finding constitutes the first report of a gene involved in the anthocyanin biosynthetic pathway and responsible for flower colour in the genus *Alstroemeria*. Nevertheless, other genes could also be involved as it has been reported that more than one gene could be related to the biosynthesis of one particular pigment [46,47].

The position of the genes *ANS* and *CHS* in the anthocyanin biosynthetic pathway could also explain the differential expression patterns observed. Thus, while the initial position of *CHS* in this pathway does not affect the biosynthesis of pigments, the location of *ANS* towards the end of the pathway could account for its direct participation in the colouration of flowers. Thus, previous studies in which *ANS* has been silenced have led to the production of white flowers of *Torenia hybrida* and pale blue gentians [48]. Furthermore, in other species such as *Forsythia suspensa* [49] and lisianthus [50], the null expression of *ANS* reduces the accumulation of anthocyanins in the petals, which shows the key role of this gene in the anthocyanin biosynthetic pathway. The development of molecular markers linked to this gene could lead to the application of molecular-assisted selection (MAS) in the breeding of this species. However, identification of other genes involved in the biosynthetic pathway of anthocyanins would also be necessary.

### 3.4. Principal Component Analysis

The principal component analysis performed demonstrates that accessions with more intense tones are associated with a higher C* value, a higher relative concentration of putative 6-hydroxycyanidin, and a higher relative expression of *ANS* compared to paler accessions (Table 3). These results suggest a correlation between phenotype, anthocyanin content and *ANS* expression for the flower colour of *A. pallida*.

In a previous study performed in *Alstroemeria* spp., a positive correlation between colour intensity and anthocyanin concentration was reported [21]. Furthermore, it was also found that hues of flowers with delphinidin 3-glycosides were bluer, and with 6-hydroxycyanidin 3-glycosides redder in comparison with flowers containing exclusively cyanidin 3-glycosides. However, correlations with the expression of key genes were not performed in this earlier study. Correlations between RHS colour chart and anthocyanins have been also performed in *Rhododendron*, categorising species into four groups (red, purplish pink, purple and white) [43]. Moreover, correlations between *CHS* expression and anthocyanin pigmentation of Oncidium orchid cultivars has been studied [51].

## 4. Materials and Methods

### 4.1. Plant Material

Flowers of *Alstroemeria pallida* (Figure 1) were analysed in situ and collected at anthesis (showing anther dehiscence) from plants growing in Farellones, Región Metropolitana, Chile (33°20′54″ S and 70°18′24″ W) in January, 2017. Samples were stored at −20 °C prior to further chemical and molecular analysis. For all subsequent evaluations (phenotype, anthocyanin content and gene expression), tepals collected from four different individuals (replicates) were considered.

### 4.2. Phenotypic Characterisation

**Figure 5:**
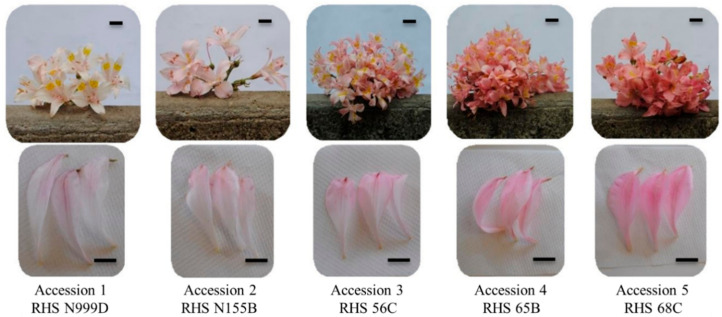
Flowers (**upper row**) and tepals (**lower row**) of five accessions of *Alstroemeria pallida*, classified by flower colour according to the RHS mini colour chart. Scale bar = 2 cm.

The flower colour of *A. pallida* was evaluated visually using an RHS mini colour chart (The Royal Horticultural Society and Flower Council Holland, London, UK). Four individuals showing the same flower colour were selected and each sample was evaluated in situ on the mid third portion of the outer tepals only, since dark marks and spots were observed on the inner tepals. This evaluation led to the identification of five different accessions ranging from white (RHS N999D) to pink (RHS 68C) (Figure 5).

Phenotypic characterisation also considered an instrumental evaluation of flower colour using standard CIELab colourspace coordinates provided by a colourimeter (Minolta, model CR-400, Osaka, Japan), programmed to use a D65 illuminant, at 0° observer and calibrated with a white standard. For each accession and replicate, three consecutive measurements were taken from the mid third portion of the outer tepals of four individuals. Values were expressed in terms of L* (lightness), C* (chroma = √ [a*]^2^ + [b*]^2^) and h* (hue = arctan [a*/b*]), where negative a* indicates green, high positive a* indicates red, high positive b* indicates yellow, and negative b* indicates blue. Moreover, in order to correlate CIELab values with the colour observed, an arbitrary value for visual colour intensity (VCI) was assigned to each accession ranging from 1 (RHS N999D, lightest) to 5 (RHS 68C, darkest).

### 4.3. Anthocyanin Characterisation

Anthocyanin extraction was performed using approximately 300 mg of tissue of *A. pallida* outer tepals mixed with 10 mL MeOH:HCO_2_:H_2_O (10:3:3) and shaken for 1 h at 175 rpm. The extract obtained was made solvent free using a rotavapor system (R-210, Buchi, Agilent 1200 series) at 35 °C. The pellet obtained was resuspended in 200 µL MeOH:H_2_O (1:1) and then filtered using a 0.22 µm millipore Millex-GV PVDF (polyvinylidene difluoride) filter. Subsequently 70 μL of the filtered extract were injected in an HPLC (Agilent Technologies, series 1200, San Diego, CA, USA.) at a flow rate of 1 mL min^−1^. Separation of the compounds was conducted using a Simetry C18 column (4.6 × 250 mm, Waters Corp.) with a particle size of 5 µm using 10% formic acid as solvent A and 10% acetonitrile as solvent B. For detection and quantification of compounds, the chromatograms were recorded at 280 and 520 nm. Anthocyanins were individually identified from each peak shown in the chromatogram produced by the HPLC using a library described previously [24], whilst the quantity of each compound was calculated as a concentration relative to malvidin 3-glucoside (Sigma, St. Louis, MO, USA). For each accession, three extractions (replicates) were performed. Finally, considering that the pH of tepal cells may affect the stability of the anthocyanins, 0.5 g tissue from each sample was ground and macerated in 5 mL double distilled water and then the pH was measured with a pH meter [5]. Each measurement was repeated four times.

### 4.4. Gene Expression

RNA was extracted from outer tepals of five accessions of *A. pallida* using the TRI Reagent method described previously [52]. DNA was removed from the samples using RQ1 DNase (Promega, Madison, Wl, USA). Subsequently cDNA was synthesised from total RNA using oligo (dT) primer and M-MLV RT enzyme (Promega).

Forward and reverse primers were designed using Primer3Plus [53] to amplify several genes involved in the biosynthesis of anthocyanins using data obtained from the Genbank database NCBI (National Center for Biotechnology Information; [54]. and from a *de novo* assembled transcriptome of *A. pelegrina* (unpublished data). Two genes were selected based on their consistent and clear amplification by PCR: *chalcone synthase* (*CHS*) and *anthocyanidin synthase* (*ANS*). For the amplification of fragments of these genes, the primers designed were: 5′-GGCTCACATTCCATCTCTTG-3′ (forward) and 5′-GATCCAGAACAGCGAGTTC-3′ (reverse) for *CHS* (257 bp), and 5′-TTCCTCCTCACCAACATGG-3′ (forward) and 5′-ACGTGCATGAACATCGAGTC-3′ (reverse) for *ANS* (95 bp).

RT-qPCR amplification was performed using an Eco real-time PCR system (Illumina, BC-100-1001, San Diego, CA, USA) with a final volume of 10 μL containing 5 μL of *Mix* SYBR *Green* (Applied Biosystems, San Diego, CA, USA), 0.25 μM of each primer and approximately 40 ng cDNA. Amplifications were carried out with the following program: 50 °C for 2 min; 95 °C for 10 min; 45 cycles of 95 °C for 15 s and 58 °C for 45 s. Real-time PCR amplification was followed by a dissociation cycle (82 °C for 5 min; 60 °C for 1 min; 95 °C for 1 min) to validate the denaturation temperature of the amplicons. Calibration curves were calculated for *CHS* and *ANS*, and for the housekeeping genes *18S* [15] and *eEF1A* [55] using 10-fold serial cDNA dilutions. The relative expression of *CHS* and *ANS* was calculated from the calibration curves and normalised against the transcript level of the housekeeping genes.

### 4.5. Statistical Analysis

Data obtained from CIELab (C*, L* and h*), anthocyanin content and gene expression were subjected to an analysis of variance (ANOVA). Means were compared using Fishers least significant difference (LSD) test for multiple pair-wise comparisons with a significance level of 0.05. Pearson correlation coefficients between the CIELab values (C*, L*, h* and VCI), anthocyanin content and gene expression were calculated. A PCA was performed to analyse the correspondences between the different components of CIELab (C*, L* and h*), VCI, anthocyanin content and gene expression. Finally, a biplot was produced using optimal scaling to perform an exploratory analysis of the relationships between the data obtained. All the statistical analyses were performed using the InfoStat software [56].

## 5. Conclusions

Floral colour is an important characteristic for breeders and consumers. The results obtained suggest a correlation between phenotype, 6-hydroxycyanidin content and *ANS* expression for the flower colour of *A. pallida*, which is of interest for further studies, mainly related to the breeding of this species with ornamental value. However, the identification and characterisation of other genes related to the biosynthetic pathway of anthocyanins and their contribution to flower colour is still needed. This information would allow breeders to perform MAS to obtain new varieties of this economically important ornamental plant, making this process more efficient when focusing on the character of flower colour. Moreover, the application of sequencing techniques could reveal allelic variation among accessions in order to select parental lines and guide hybridisation of this species to obtain new varieties. Thus, the identification of the first gene associated with flower colour in this species is an important contribution to continue the research on this key characteristic for alstroemeria as an ornamental plant.

## Figures and Tables

**Table 1:** Characterisation of the flower colour of five *A. pallida* accessions including code and colour according to the RHS mini colour chart, and C*, h* and L* values measured with a colourimeter. An arbitrary scale of visual colour intensity (VCI) is also included. Means of 12 replicates are shown, and different letters indicate significantly different values (analysis of variance (ANOVA), *p* < 0.05).

Accession	Colour	VCI	C*		h*		L*	
RHS N999D	White	1	8.72	d	72.55	a	87.02	a
RHS 155B	White	2	9.76	cd	34.25	b	83.77	ab
RHS 56C	Light Pink	3	10.34	c	31.86	b	81.19	b
RHS 65B	Light Pink	4	17.40	b	20.78	c	81.50	b
RHS 68C	Pink	5	22.02	a	12.54	d	76.26	c

**Table 2:** Pearson correlations (r) between different variables related to flower colour of five accessions of *A. pallida*.

Variables	r
L*/C*	−0.76 ^†^
L*/h*	0.75 ^†^
L*/VCI	−0.81 ^†^
C*/h*	−0.70 ^†^
C*/VCI	0.91 ^†^
H*/VCI	−0.86 ^†^

^†^ Correlation significant at *p* ≤ 0.05.

**Table 3:** Correlations between L*, C*, h*, VCI, 6-hydroxycyanidin relative concentration and *CHS* and *ANS* gene expression, observed in the principal component analysis for the five *A. pallida* accessions (Significance\Correlation).

	Correlation	L*	H*	C*	VCI	6-Hydroxycyanidin Concentration	*CHS* Expression		*ANS* Expression	
Significance							Ref.18S	Ref.eEF1A	Ref.18S	Ref.eEF1A
L*			0.74	−0.75	−0.81	−0.36	0.16	0.22	−0.56	−0.37
H*		*		−0.72	−0.89	−0.54	0.11	−0.001	−0.54	−0.58
C*		*	*		0.92	0.11	0.03	0.06	0.66	0.70
VCI		*	*	*		0.26	−0.06	−0.004	0.58	0.65
6-hydroxycyanidin concentration		ns	*	ns	ns		0.08	0.23	0.45	0.35
*CHS* Ref. 18S		ns	ns	ns	ns	ns		0.80	0.46	0.22
*CHS* Ref. eEF1A		ns	ns	ns	ns	ns	*		0.33	0.48
*ANS* Ref. 18S		*	*	*	*	*	*	ns		0.59
*ANS* Ref. eEF1A		ns	*	*	*	ns	ns	*	*	

ns = Correlation not significant (ANOVA followed by an LSD test); * = Correlation significant (ANOVA followed by an LSD test, *p* ≤ 0.05).

**Figure A1:**
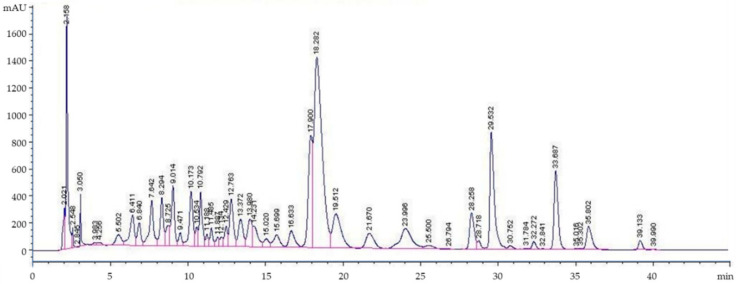
Chromatogram produced by HPLC at 280 nm of the analysis of *A. pallida* outer tepals expressed in milli-absorbance unit (mAU).

## Appendix A

**Table A1:** Tepal cell pH measured on five *A. pallida* accessions.

Accession	Tepal Cell pH
RHSN999D	5.00 a
RHS155B	5.01 a
RHS56C	5.01 a
RHS65B	5.01 a
RHS68C	4.99 a

Different letters indicate significant differences using ANOVA followed by an LSD test (*p* ≤ 0.05).
